# Benefits and durability of an affordable prosthetic silicone cushion liner locally manufactured in a resource-limited environment: Enabling adoption of total surface bearing sockets and silicone cushion liners

**DOI:** 10.1097/PXR.0000000000000433

**Published:** 2025-01-22

**Authors:** Claudia Ghidini, Shova Kanta Sharma, Saloman Shrestha, Dhan Prasad, Clément D. Favier, Jeffrey Erenstone, Anthony M. J. Bull, Suraj Maharjan

**Affiliations:** 1 Department of Bioengineering, Imperial College London, London, UK; 2 Green Pastures Hospital and Rehabilitation Centre, Pokhara, Nepal; 3 Operation Namaste, Lake Placid, US

**Keywords:** prosthetic care, silicone liners, resource-limited environments, below-knee amputation, mobility, transtibial

## Abstract

**Introduction:**

Prosthetic silicone liners improve comfort and skin protection and allow the use of total surface bearing (TSB) sockets, which provide enhanced proprioception and comfort. Unfortunately, silicone liners are cost-prohibitive in resource-limited environments (RLEs) where patellar tendon bearing (PTB) sockets with PE-lite liners remain standard, leading to patient discomfort and skin issues.

**Objective:**

This study evaluates the benefits and durability of an affordable silicone liner locally manufactured in an RLE to promote TSB socket adoption, aiming to enhance prosthetic care and patient outcomes.

**Methods:**

Ethical approval was granted by the Nepal Health Research Council. Twelve people with a unilateral transtibial amputation who were using a PTB socket with PE-lite participated in the study and received a new prosthesis (TSB socket with an affordable silicone liner). Participants performed mobility tests (2-Minute Walking Test, Timed Up and Go Test) and completed self-reported questionnaires for both prostheses. Liner durability was assessed.

**Results:**

Participant mobility improved while wearing the TSB socket and silicone liner. The new prosthesis was found to be comfortable, and there were no major problems identified although excessive sweating, typical with silicone liners, was reported. Liners were replaced after 6.1 (±3.1) months.

**Conclusion:**

A locally manufactured liner provided increased mobility and high levels of satisfaction. This affordable liner may be suitable for use in RLEs, enabling adoption of TSB sockets and improving rehabilitation outcomes of people with a transtibial amputation. However, durability concerns and excessive sweating would suggest that improvements can still be made.

## Introduction

More than 600 million people in the world have a disability, and 80% of those live in low- and middle-income countries (LMICs) and resource-limited environments (RLEs).^1,2^ While about 1.5 million people undergo amputations every year, the World Health Organization estimates that only 5-15% of people with amputation who need prosthetic devices in LMICs and RLEs have access to them.^3^ High prices of prosthetic devices in LMICs, combined with high indirect costs for users (e.g., travel),^3^ make prosthetic services unaffordable to many of the people who need them. Basic prosthetic devices, most commonly available in LMICs, can cost from USD 500 up to USD 2,000, whereas more advanced components can range from USD 15,000 up to USD 70,000.^3^

Prosthetic silicone liners are products that roll directly onto the residual limb and improve prosthesis function by enhancing cushioning and skin protection.^4^ There are 2 primary types of silicone liners: cushion and locking liners. While both enhance comfort and promote residual limb health, locking liners, equipped with a pin and locking mechanism within the socket, additionally offer suspension for the prosthesis. Unfortunately, silicone liners are part of the cost-prohibitive components for RLEs.^3,5^ They are not locally available and must be purchased from abroad, costing from USD 200 to 500 per liner.^3,5^ The lack of local alternatives partly contributes to the high cost of these products and hinders accessibility because of supply chain challenges present in LMICs.^3,5^ In addition, silicone liners are usually replaced annually and this high rate of replacements makes them even less accessible in RLEs where resources are stretched and replacement should be minimized.^3^

In high-resource environments where silicone liners are easily accessible, they are widely provided to most of people with amputations because of their numerous benefits, including improved residual limb health and comfort.^3,6–8^ In addition, silicone liners facilitate the use of transtibial (TT) total surface bearing (TSB) sockets, a design developed in the 1990s when silicone liners were first introduced.^3,9^

TSB sockets improve proprioception, comfort, and control of the prosthesis because the weight is borne uniformly across the residual limb^7,8,10^ although it is important to note that TSB sockets do not apply pressure on bony prominences such as the fibular head. Most people with a TT amputation in RLEs are still using patellar tendon bearing (PTB) sockets where the weight is borne on the patellar tendon, creating a high pressure point,^8^ as well as other weight-bearing areas.

This high pressure combined with the use of liners made of ethylene-vinyl acetate sponge foam, known as PE-lite liners, leads to a higher prevalence of pain and skin conditions like ulcers.^3,8^ These residual limb conditions are exacerbated for people with leprosy, still common in Nepal.^11^ Leprosy is a chronic infection caused by *Mycobacterium leprae* and is primarily a disease of peripheral nerves, skin, and mucosa, which can often lead to loss of sensation.^11,12^ Therefore, leprosy-affected people are more likely to experience severe ulceration and damage to the soft tissues with PTB sockets and require high-quality care and prosthetic components.^13^ Widespread leprosy further underscores how access to silicone liners is of utmost importance in Nepal because it is a key factor to ensure appropriate rehabilitation and avoid secondary complications.^14^ The lack of an economically viable silicone or elastomeric gel liner is currently the greatest barrier to access in RLEs.^3^

In response to a critical need identified by the global partnership for Assistive Technology, Assistive Technology Scale,^3^ the aim of this research study is to assess the benefits and durability of an affordable silicone cushion liner, locally manufactured in Nepal, to enable adoption of TSB sockets in RLEs. As part of this study, objective and perceived functional mobility and overall satisfaction are compared between prostheses with this affordable silicone liner and standard prostheses currently available in most RLEs.

## Methods

### Workshop setup

Computer-aided designed liner molds were 3D-printed in the United States and the United Kingdom and shipped to Kathmandu, Nepal, along with medical grade, platinum-cured silicone (shore hardness 00:60)^15^ to set up a workshop on-site. An iterative approach was used to arrive at this shore hardness to balance comfort and durability. Computer-aided designed files of molds were scaled to 5 liner sizes to accommodate individuals with different residual limb sizes. The sizes are selected measuring the residual limb circumference 5 cm from the distal end, and the sizes are size XS for circumference 14.5–17.5 cm, size S for circumference 18–21.5 cm, size M for circumference 22–25 cm, size L for circumference 26.5–31 cm, and size XL for circumference 31.5–36.5 cm. A local team was trained on the process of making the cushion liners. Standard operating procedures were also provided, including final checks to guarantee product quality (e.g., checking for the absence of bubbles in the cured silicone).

### Cushion liner fabrication

**Figure 1.**
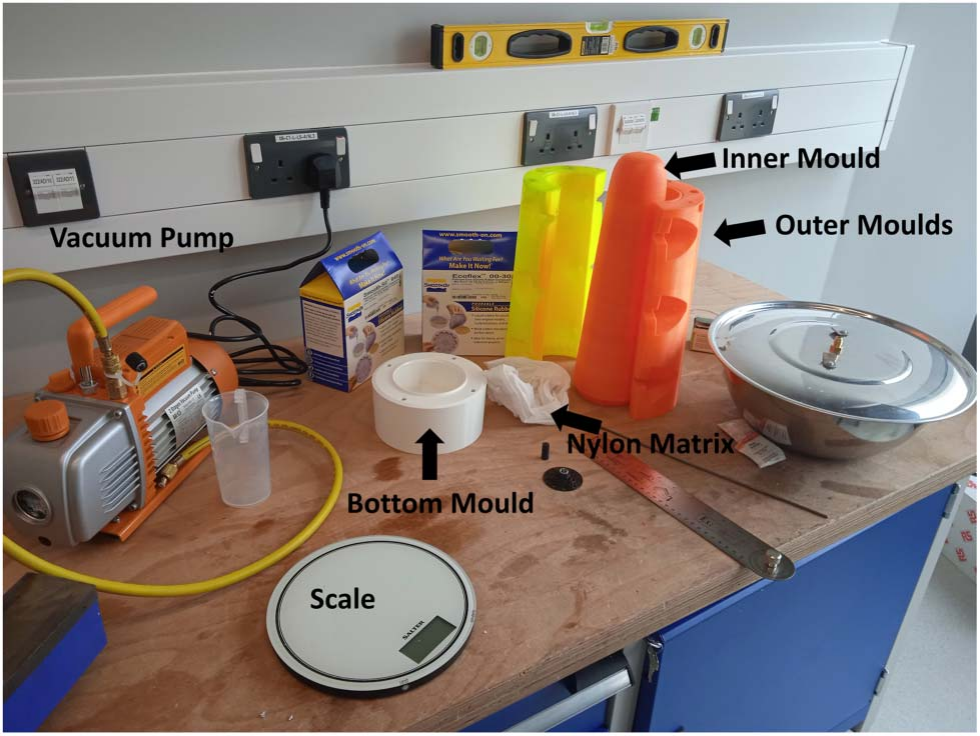
Equipment to manufacture a cushion silicone liner locally: 3D-printed inner and outer molds, vacuum pump, scale, silicone, nylon matrix.

After selection of the appropriate size, outer molds are chosen and assembled with bolts and nuts (Figure 1). A nylon matrix is sleeved into the inner mold, which is then inserted in the assembled molds before the silicone is poured. Parts A and B of a platinum medical grade cured silicone are mixed in a 1:1 ratio and degassed using a vacuum pump and a vacuum chamber^16,17^ with a pressure of −100 kPa. To ensure proper degassing, bubbles should surface and explode and the degassing procedure should be repeated twice.^15^ The mixed, degassed silicone is then poured into the molds and left to cure with the nylon matrix for 24 hours. As commercially available silicone liners are often fabricated with an outer fabric to enhance durability, a fabric is glued to the outside of the liner and left to cure for another 24 hours. This fabric is glued with an adhesive sealant designed for bonding silicone rubber.^18^ The liner manufactured with these molds has a tapered profile, which is thicker at the distal end for improved comfort (11 mm) and thinner at the proximal end for ease of donning/doffing (2.5 mm). To select the appropriate size for the liner and molds to be used, the circumference of the residual limb is measured 5 cm from the distal end and a sizing chart is used.

The cost of the raw materials to fabricate a liner is approximately $60 (at the point of manuscript submission; silicone, sleeve, outer fabric, 3D printing filament for molds, screws, and nuts). This does not include cost of the equipment needed that would amortize over time and with production scale-up (3D printer, vacuum pump), labor cost, and shipping/custom cost for importing silicone.

The molds, bill of materials, and detailed manufacturing guidelines can be obtained from Operation Namaste by submitting a request on their website.^19^

### TSB training

As PTB sockets are the standard sockets provided by Green Pastures Hospital and Rehabilitation Centre (GPHRC), the 2 prosthetists involved in the research study (SKS, DP) received refresher training in the rectification process of TSB sockets. The training was blended, included both theory and hands-on practice, and was developed by Operation Namaste Education Team.^19^ The training was included to ensure that the quality of the prosthesis and participant's safety and comfort were prioritized. The training lasted 1 day and was delivered at GPHRC. At the end of training, 2 prosthetic sockets were made and fitted to ensure that the process was clear and any additional doubt could be clarified.

### User trial

A user trial was designed to investigate durability of silicone liners and changes in functional mobility and to collect users' opinion. Ethical approval was granted by the National Research Ethics Committee of Nepal (Study Number: 54/2022 P) on April 27, 2022, and the research study was conducted following the guidelines for the delivery of research in RLEs.^20^ Local researchers (SKS, DP), who are also the treating prosthetists of the participants, screened the list of current prosthetic users at GPHRC to identify suitable candidates and invite them to participate in the research study. Seventeen people with a TT amputation who were currently using a PTB self-suspended socket (supracondylar suspension) with PE-lite liner were recruited as part of the study at GPHRC (Pokhara, Nepal) and provided written informed consent. GPHRC was established in 1957 to provide treatment and rehabilitation for people with leprosy and people with other physical disabilities,^21,22^ and it is one of 5 key facilities providing specialized prosthetic rehabilitation in Nepal.^23^

Participants were provided with a new prosthesis. The new prosthesis consisted of socks, the locally manufactured silicone liner, a TSB self-suspended socket, standard polypropylene pylon and connecting part, and a solid-ankle-cushion-heel (SACH) foot. The cost of the prosthesis, $350, and the cost of the silicone liners necessary for the lifespan of the prosthesis (∼3-5 years), $300, were covered as part of the research study. The participant's usual current prosthesis, used as comparison in this research study, consisted of socks, a PE-lite liner, a PTB self-suspended socket, standard polypropylene pylon and connecting part, and an SACH foot. The differences between the new prosthesis and their current one were the use of the silicone cushion liner and the socket type.

Participants were trained in caring for, and use of, their silicone liner including washing and donning and doffing. Participants were also given an informative leaflet. Participant's demographics and information on the amputation were collected.

Participants performed validated mobility tests^24,25^ for their current prosthesis and new prosthesis that included the cushion liner. The mobility tests chosen were informed by the recommendations of Consensus Outcome Measures for Prosthetic and Amputation Services^26^ and consisted of the 2-Minute Walking Test (2MWT) and Timed Up and Go Test (TUGT). These tests were performed for the current prosthesis at recruitment and for the new prosthesis at follow-up (>3-month usage). The 2MWT consists of walking for 2 minutes on a straight, even 20-m track with a sign on the floor to mark the turning point.^26^ Mean walking speed was calculated as distance walked over time taken. An increase in the distance walked indicates an improvement in mobility. The TUGT consists of standing up from a chair, walking 3 m, turning around, walking back, and sitting down again.^26^ Participants are timed for this test, which is repeated twice as per the test protocol.^26^ The faster of the two-timed trials is selected.^26^ A lower time indicates better mobility.

Participants completed a validated self-reported questionnaire, Prosthetic Limb Users Survey of Mobility (PLUS-M) 12-item Short Form,^27^ to assess the perceived change in mobility for their usual PTB socket and the new TSB socket with silicone liner. The questionnaire was completed at recruitment for their current prosthesis and over the phone after 3 months of using the new prosthesis. The questionnaire was chosen because it has been validated with >1,000 unilateral lower-limb prosthetic users and it is available in Nepali.^27,28^ The questionnaire consists of 12 questions where participants are asked about their ability to perform daily activities that require use of the lower limbs, ranging from household ambulation to outdoor recreational activities.^27^ The score goes from 5 (perfectly able to) to 1 (unable to do it).^27^ The raw total score is obtained by summing all the scores associated with each answer and is then converted to total T-score following the PLUS-M conversion table.^27^ T-Scores of the full development cohort and subgroups, including people with TT amputation and different causes of amputation, are provided in the PLUS-M User Guide.^27^

In addition, the Satisfaction with Prosthesis (SAT-PRO) questionnaire^29^ was tailored to the scope of this research study. Some statements were adapted or new statements were added to ask about problems generally encountered with silicone liner such as excessive sweating^30^ and compression, whereas items not pertinent to the scope of the research study were removed (e.g., “My prosthesis is easy to understand”). This questionnaire was completed over the phone at a 3-month follow-up. Although questions were changed, the total score was calculated following the guidelines published by the authors of the SAT-PRO.^29^ In the SAT-PRO, each statement is rated from 3 (completely agree) to 0 (completely disagree).^29^ The answers to different statements of all participants are then averaged and reported as percentage of total score, where 3 is the maximum score for each answer. The total average score of the questionnaire was also calculated, and the maximum attainable score is 30, which represents the highest satisfaction with the prosthesis and liner. Participants were also contacted over phone after 2 and 4 weeks to check how they were adapting to the new prosthesis.

A summary of the data collected for each prosthesis and methods is provided in Table 1.

**Table 1.**
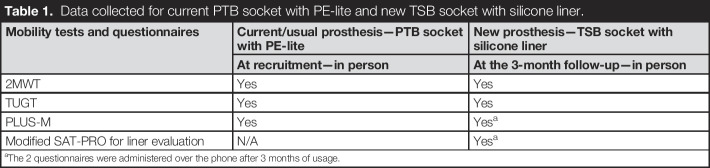
Data collected for current PTB socket with PE-lite and new TSB socket with silicone liner.

Mobility tests and questionnaires	Current/usual prosthesis—PTB socket with PE-lite	New prosthesis—TSB socket with silicone liner
	At recruitment—in person	At the 3-month follow-up—in person
2MWT	Yes	Yes
TUGT	Yes	Yes
PLUS-M	Yes	Yes^a^
Modified SAT-PRO for liner evaluation	N/A	Yes^a^

a The 2 questionnaires were administered over the phone after 3 months of usage.

Finally, durability of the new liner was assessed based on the number of months before a replacement was needed.

### Statistical analysis

Descriptive statistical methods were used. Inferential statistical methods were used to explore differences in results of mobility tests and PLUS-M questionnaire between 2 groups (PTB socket with PE-lite, TSB socket with silicone liner at follow-up). Normality of the distributions was assessed using the Shapiro–Wilk test for continuous variables.^31^ If the distribution was found to be normal, the independent-samples t-test was run for normal distributions; otherwise, the Mann–Whitney U Test was used.^31^

## Results

Seventeen participants were recruited. Five participants did not return to collect the new prosthesis or were lost to follow-up, so results are presented for 12 participants who completed the full study including the 3-month follow-up (Table 2). Participants were prevalently young males (75.0%) who lost their limb at a young age (25.1 ± 16.8 years of age), with trauma being the most common cause of amputation (58.3%). Participants' mean age at the time of recruitment was 36.9 ± 14.2 years.

**Table 2.**
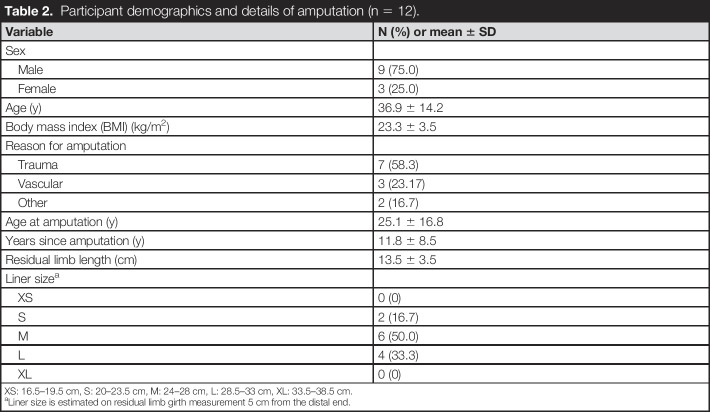
Participant demographics and details of amputation (n = 12).

Variable	N (%) or mean ± SD
Sex	
Male	9 (75.0)
Female	3 (25.0)
Age (y)	36.9 ± 14.2
Body mass index (BMI) (kg/m^2^)	23.3 ± 3.5
Reason for amputation	
Trauma	7 (58.3)
Vascular	3 (23.17)
Other	2 (16.7)
Age at amputation (y)	25.1 ± 16.8
Years since amputation (y)	11.8 ± 8.5
Residual limb length (cm)	13.5 ± 3.5
Liner size^a^	
XS	0 (0)
S	2 (16.7)
M	6 (50.0)
L	4 (33.3)
XL	0 (0)

XS: 16.5–19.5 cm, S: 20–23.5 cm, M: 24–28 cm, L: 28.5–33 cm, XL: 33.5–38.5 cm.

a Liner size is estimated on residual limb girth measurement 5 cm from the distal end.

**Figure 2.**
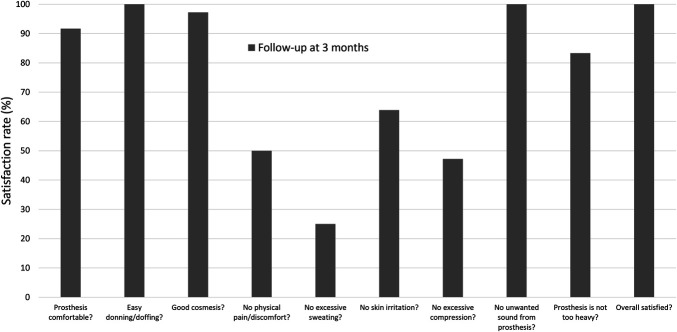
Liner satisfaction ratings at the 3-month follow-up using the modified SAT-PRO (n = 12). 100% means that all participants were fully satisfied with the item, whereas 0% means that none of the participants were satisfied.

At the 3-month follow-up, there was a significant increase in distance of ambulation (154 ± 21 m vs 134 ± 19 m; *p* = 0.026) and speed (1.28 ± 0.17 m/s vs 1.12 ± 0.16 m/s; *p* = 0.005) while wearing the TSB socket with the affordable silicone liner compared with the PTB socket with PE-lite. There was no difference in TUGT (PTB with PE-lite: 9.70 ± 2.17 s, TSB with silicone liner: 8.46 ± 1.22 s; *p* = 0.114). There was a trend for an increase in self-reported perceived mobility as shown by the T-score of the PLUS-M (PTB with PE-lite: 46.5 ± 5.3, TSB with silicone liner: 54.4 ± 7.5; *p* = 0.056), which reached close to the average T-score of individuals with traumatic TT amputation provided by the PLUS-M guidelines (mean T-score = 55.9, n = 338).^27^ Overall satisfaction with the liner was high (Figure 2). The new prosthesis was found to be comfortable; there were no problems identified with donning and doffing the liner, nor with product maintenance or cosmetic appearance. Participants were the least satisfied with the following problems caused by wearing the silicone liner: physical pain and discomfort, sweating problems, skin irritation, and excessive compression.

**Figure 3.**
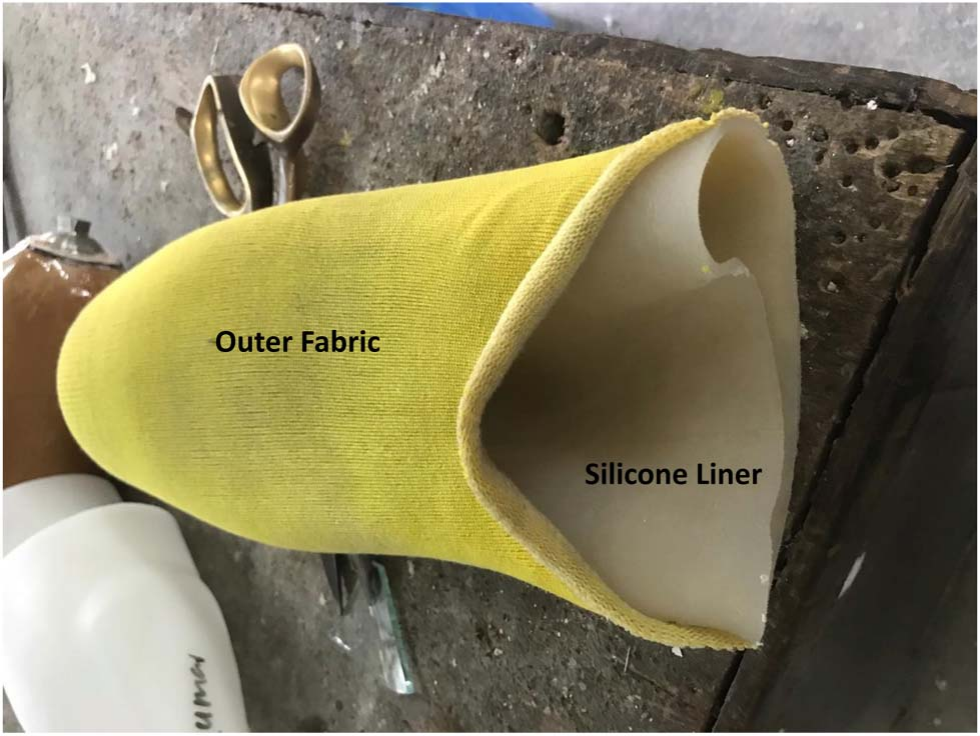
Common failure mode: detachment of the outer fabric.

The lifespan of the novel silicone cushion liner was 6.1 months (±3.1). The following failure modes were identified: torn silicone inside, torn inner nylon matrix, and detachment of the outer fabric at the proximal end of the liner (Figure 3).

## Discussion and conclusion

The study focuses on a cohort of young working-age individuals who have experienced limb loss primarily because of trauma, with no significant other comorbidities. The participants were significantly younger than the typically older, vascular/diabetic amputees in high-resource settings.^32,33^ Access to appropriate prostheses and care is crucial to ensure full rehabilitation of young and active people to enable them to contribute to their family and community.^3^

The new liner and socket led to increased mobility as evidenced by both objective assessments and patient-reported outcome measures, including almost matching the benchmark level of perceived ability to perform daily activities (mean T-score). Three months was selected as an appropriate period to allow participants to acclimatize to the new prosthesis, including adjustments to increased compression resulting from both the liner and the TSB socket design which compress the residual limb uniformly. It is not known if a longer period would result in further improvements. Although 5 liner sizes were provided, only the 3 middle sizes were used, suggesting a potential rationalization, in this setting, of the equipment required.

There were challenges with the new liner/socket system, including issues such as excessive compression, skin irritation, discomfort, and sweating. Although sweating is a commonly reported issue by silicone liner users,^30^ this issue might pose an additional challenge in humid climates like in Nepal. Addressing this issue by modifying liner design is imperative for enhancing user satisfaction and ensuring optimal prosthesis utilization. In addition, durability is identified as a critical technical requirement in prosthetic components, particularly in RLEs where resources are constrained.^2,3,34^ This study highlighted concerns regarding liner durability, including tears at the distal end and detachment of the outer fabric after an average 6-month usage. Different options should be explored to increase the liner's lifespan including using alternative materials or using different gluing methods. Ensuring durability is essential not only for prolonging the lifespan of prosthetic components but also for minimizing the burden on healthcare resources in RLEs.^2,3^ Therefore, future design considerations should prioritize durability enhancements to optimize the effectiveness and sustainability of novel prosthetic interventions for these settings.

The study's significance extends beyond its specific findings because it represents the first investigation involving locally manufactured silicone cushion liners within RLEs. The importance of trialing new devices in intended settings is emphasized because laboratory trials may overlook critical factors such as user lifestyles (i.e., agricultural or physical labor use cases) and durability when in harsher environmental conditions.^3^ In addition, inclusion of the clinical team from the beginning is deemed essential for ensuring research appropriateness because clinicians are familiar with the culture, environment, patient-specific needs, and resources available.^20^ Their inclusion from the beginning can improve the chances of success of the study. For instance, they recommended choosing simple tests that could be delivered without the need for additional and costly equipment and embedded in the processes of the clinic to facilitate the patient journey. These recommendations have also been supported by research teams working in RLEs.^35^ This is the reason behind the selection of the 2MWT and TUGT which could be successfully deployed anywhere with no extra equipment cost or requiring additional human resources.^26^

There are some limitations to this study, including the cross-sectional nature of the chosen mobility tests (2MWT, TUGT), which do not fully capture daily usage and activity levels. Future research could explore longitudinal activity monitoring to assess the impact on activities of daily living in real-life home use settings. In addition, although an improvement compared with PE-lite liner was found, comparative studies with commercially available products would enable a comparison with existing (higher cost) options. Furthermore, some of the study participants had been using PTB socket with PE-lite for decades and might have experienced difficulties in adapting to the new prosthesis. Exploring primary users' perspectives to evaluate satisfaction levels and pinpoint additional areas requiring improvement represents another avenue for future research. In addition, TSB sockets are typically used with a sleeve or seal to improve suspension, but these components are not commonly available in RLEs. The study aimed to test the device in its intended environment with the available resources, so the TSB socket was used with self-suspension only, without the additional sleeve. This reduced suspension might have influenced the results. However, not using the sleeve minimized the number of variables changed, allowing the study to focus solely on assessing the benefits of the liner when used with the TSB socket. Furthermore, this study did not include prosthetic users with leprosy because appropriate sensation in the residual limb was necessary to provide feedback on physical comfort. Following appropriate design updates, future research should investigate benefits of this silicone liner with leprosy patients. Finally, the modified SAT-PRO questionnaire used in this study has not been validated. The original version treats the prosthesis as aunique system, failing to identify challenges related to individual components such as prosthetic foot or silicone liner. To address this, a modified version was necessary to identify specific design issues with liner. However, the modification might have introduced potential biases because the authors selected new questions and excluded some existing ones that were not relevant to the research objectives. The added questions were informed by a literature review identifying common problems with liner usage. No existing questionnaire specifically evaluates liners, so the SAT-PRO had to be adapted.

In conclusion, the study shows that a lower-cost locally manufactured silicone cushion liner and TSB socket may be successfully and safely used in RLEs, provided that few specific design updates are made. This is the first study that has trailed a locally manufactured silicone liner in its intended environment. The research highlights the pivotal role appropriate prosthetic components can play in facilitating the rehabilitation of young and active individuals and aiding their return to work and social life. Improving care for people with lower-limb amputation through the availability of diverse prosthetic options which were designed for—and tested in—the intended environment is imperative for enhancing their quality of life and societal inclusion.

## Funding

The author(s) disclosed receipt of the following financial support for the research, authorship, and/or publication of this article: This work was supported by the Impact Accelerator Account grant (PSM837) from the UK Engineering and Physical Sciences Research Council.

## Declaration of conflicting interest

JE is one of the founders and president of Operation Namaste, the charity that provided the locally manufactured silicone liners. This product is not yet approved by the regulatory bodies, and it is still investigational. The other authors have no conflict of interest to declare.

## Supplemental material

No supplemental digital content is available in this article.
